# Precision Medicine for Neonatal Sepsis

**DOI:** 10.3389/fmolb.2018.00070

**Published:** 2018-07-26

**Authors:** Sherrianne Ng, Tobias Strunk, Pingping Jiang, Tik Muk, Per T. Sangild, Andrew Currie

**Affiliations:** ^1^Medical and Molecular Sciences, School of Veterinary and Life Sciences, Murdoch University, Perth, WA, Australia; ^2^Centre for Neonatal Research and Education, The University of Western Australia, Perth, WA, Australia; ^3^Department of Veterinary and Animal Sciences, University of Copenhagen, Frederiksberg, Denmark

**Keywords:** systems biology, diagnosis, infection, neonate, sepsis, preterm infant

## Abstract

Neonatal sepsis remains a significant cause of morbidity and mortality especially in the preterm infant population. The ability to promptly and accurately diagnose neonatal sepsis based on clinical evaluation and laboratory blood tests remains challenging. Advances in high-throughput molecular technologies have increased investigations into the utility of transcriptomic, proteomic and metabolomic approaches as diagnostic tools for neonatal sepsis. A systems-level understanding of neonatal sepsis, obtained by using omics-based technologies (at the transcriptome, proteome or metabolome level), may lead to new diagnostic tools for neonatal sepsis. In particular, recent omic-based studies have identified distinct transcriptional signatures and metabolic or proteomic biomarkers associated with sepsis. Despite the emerging need for a systems biology approach, future studies have to address the challenges of integrating multi-omic data with laboratory and clinical meta-data in order to translate outcomes into precision medicine for neonatal sepsis. Omics-based analytical approaches may advance diagnostic tools for neonatal sepsis. More research is needed to validate the recent systems biology findings in order to integrate multi-dimensional data (clinical, laboratory and multi-omic) for future translation into precision medicine for neonatal sepsis. This review will discuss the possible applications of omics-based analyses for identification of new biomarkers and diagnostic signatures for neonatal sepsis, focusing on the immune-compromised preterm infant and considerations for clinical translation.

## Introduction

Neonatal sepsis, a bacterial bloodstream infection associated with inflammation and life-threatening organ dysfunction, is classified as early-onset sepsis (EOS, < 72 h after birth) or late-onset sepsis (LOS, >72 h) (Bateman and Seed, 2010). Prompt and accurate diagnosis based on clinical and laboratory findings remains challenging. The complex and dynamic disease pathophysiology often results in clinical signs that are subtle, non-specific and overlap with non-infectious conditions (Camacho-Gonzalez et al., 2013). Consequently, there is no consensus definition for neonatal sepsis (Wynn, 2016). Further, the current “gold standard” test, microbiological culture, lacks sensitivity and has slow turnaround times (24–48 h). Adjunct tests such as hematological indices and inflammatory markers either have poor sensitivity and specificity or require serial measurements (Delanghe and Speeckaert, 2015). Simple, rapid and accurate diagnostic tests that can guide treatment of septic infants are urgently needed (Dong and Speer, 2015). Markers with high negative predictive value may allow empiric antibiotic treatment of uninfected infants to be withheld and reduce unnecessary antibiotic exposure associated with increased adverse short- and long-term outcomes (Kuppala et al., 2011; Arboleya et al., 2015).

Neonatal sepsis pathophysiology involves multiple organ systems; highlighting the need for a systems biology approach to capture the complex interactions between biological systems during disease (Smith et al., 2014; Alyass et al., 2015). Advances in genomics, transcriptomics, proteomics and metabolomics inform us of the genetic predispositions to sepsis; transcriptional changes in host responses during sepsis; protein expression altered by sepsis; and metabolites produced as a result of sepsis (Fanos et al., 2013). Integrating findings from these tools into our understanding of disease pathophysiology will enable future translation into precision medicine, where patients are identified and treated based on genetic, cellular and molecular markers that relate to the underlying causes of their disease instead of common phenotypic signs of sepsis (Flores et al., 2013; van Karnebeek et al., 2018).

This review discusses the potential of current “omics” approaches to characterize sepsis pathophysiology and allow the discovery of new biomarkers and neonatal sepsis signatures. The necessary considerations for translating these omics-based approaches from bench-to-bedside are also considered.

### Transcriptomics of the host response to sepsis

Changes in hematological markers during bacterial infections, such as in immature-to-total neutrophil ratios and white blood cell (WBC) counts, have been used for decades as adjunct tests for neonatal sepsis diagnosis. However, the clinical utility of tests remains limited by wide ranges of specificity (31–100%) and sensitivity (17–90%), especially early in sepsis onset, and by the considerable influence of common variables such as gestational and postnatal age (Schmutz et al., 2008; Chirico and Loda, 2011; Polin, 2012; Sharma et al., 2017).

The introduction of microarrays and next-generation sequencing (NGS) technologies, particularly RNA-Sequencing (RNA-Seq), has transformed our ability to monitor gene-expression changes occurring at cellular level during sepsis. Transcriptional profiling allows simultaneous measurement of expression levels of thousands of genes, where differentially expressed genes associated with sepsis could lead to the discovery of novel cell-specific gene signatures for early and accurate diagnosis of septic infants. Additionally, visualization of gene interaction networks and identification of enriched pathways associated with disease could improve our understanding of the relationship between innate, adaptive and metabolic responses during neonatal sepsis (Chaussabel et al., 2010; Skibsted et al., 2013; Smith et al., 2014; Xia et al., 2015). This section focuses on transcriptomic sepsis studies in the neonatal population (Table 1).

**Table 1:** Summary of studies using transcriptomics, proteomics and metabolomics approaches.

**Reference**	**Methodology**	**Study population and case definitions**	**Type of sample**	**Principal findings**	**Study limitations**
Smith et al., 2014	Microarray with 48,802 human gene probes	62 preterm and term neonates (24–42 weeks gestation); infected (*n* = 27) and controls (*n* = 35) **Infected cases** •Confirmed positive blood culture for Gram-negative or Gram-positive bacteria. •Full clinical assessment for early and late signs and symptoms of sepsis (e.g., presence of lethargy, jaundice, temperature instability, bradycardia and abnormal lab parameters for white cell count, neutrophil count and platelets) **Controls** •Blood collected for non-clinical reasons (e.g., screening test for maternal thyroid disease, bilirubin check for jaundice or electrolyte checks)	Peripheral blood	•Identified a 52-gene immune-metabolic network associated with sepsis •The individual innate, adaptive and metabolic pathway markers had accuracy of 84, 65, and 74%, respectively; combined use of three pathway markers had the highest accuracy of 98% for predicting bacterial infections in neonates	•The robustness of the 52-gene immune-metabolic network for predicting bacterial infections not yet validated in large-scale studies across multiple neonatal units
Cernada et al., 2014	Microarray with >28,000 human gene probes	36 VLBW infants; septic (*n* = 17) and controls (*n* = 19) **Septic cases** •Positive blood culture for Gram-positive or Gram-negative bacteria •Presence of risk factors (e.g., maternal chorioamnionitis, mothers incompletely treated or not tested for group B streptococcus infection and/or exposure to indwelling devices or surgery) •Presence of >3 clinical signs (e.g., temperature instability, respiratory symptoms including apnea or cyanosis, tachycardia or bradycardia, neurological symptoms including hypotonia or lethargy and/or gastrointestinal symptoms including vomiting or poor feeding) **Controls** •Blood collected from infants without clinical signs of infection	Peripheral blood	•Genome-wide expression profiles could discriminate between septic infants and controls with 100% sensitivity and 68% specificity •Showed 554 genes were differentially expressed between neonates with bacterial sepsis and matched controls, with 66 genes associated with tumor necrosis factor and 56 genes with cytokine signaling	•Limited number of patients in the study •Authors consider study as “starting point to perform strongly powered, prospective collaborative studies in the neonatal population”
Wynn et al., 2015	Microarray with 20,533 human gene probes	68 preterm and term neonates (23–42 weeks gestation; with EOS (*n* = 6), LOS (*n* = 9), clinical sepsis (*n* = 22) and uninfected (*n* = 31)	Peripheral blood	•Identified significant differences in transcriptome of infants with EOS or LOS; and showing importance of accounting for timing of sepsis episode when investigating transcriptional profiles	•Limited sample size especially for EOS and LOS groups •Whole blood analyses without cell typing. This limits examination of cell-specific gene expression
		**Sepsis (EOS/LOS) cases** •Positive blood culture for Gram-positive or Gram-negative bacteria •Presence of persistent (>2 days) abnormal clinical signs such as ill appearing and respiratory or cardiovascular signs •Presence of abnormal laboratory results showing systemic inflammation (e.g., CRP >45 mg/L within 48 h of evaluation) **Clinical sepsis cases** •Negative blood culture •Presence of persistent (>2 days) abnormal clinical signs such as ill appearing and respiratory or cardiovascular signs •Presence of abnormal laboratory results showing systemic inflammation (e.g., CRP >45 mg/L within 48 h of evaluation) **Uninfected cases** •Negative blood culture •Discontinued antibiotics treatment < 48 h after initiation. •CRP < 10 mg/dL in at least two of the serial measurement results with 24 h apart			•Minimal mortality in cohort limited comparisons between survivors and nonsurvivors
Chen et al., 2014	miRNA microarray and qRT-PCR	48 neonates; preterm (< 37 weeks gestation, *n* = 5) and term (>37 weeks gestation, *n* = 43); with Gram-positive (*n* = 12), Gram-negative sepsis (*n* = 12) and uninfected (*n* = 24) **Sepsis cases** •Positive blood culture with Gram-positive or Gram-negative bacteria, respectively •Positive clinical or laboratory screen **Uninfected cases** •Negative blood culture •Negative clinical and laboratory screen	Peripheral blood	•Identified significant up-regulation of miR-101/122/185 and down-regulation of miR-96/182/141/143/181a/29a/1184 in infants with neonatal sepsis •Significantly altered miRNAs identified were involved in host immune responses (e.g., pathogen recognition, pro-inflammatory cytokine release and immune cell activation) during neonatal sepsis	•Exploratory pilot study based on available clinical samples with no formal power or sample size calculations •Limited sample size with no age-matched controls
Wang et al., 2015	qRT-PCR	87 term neonates; with sepsis (*n* = 46) and controls (*n* = 41) **Sepsis cases** •Positive blood culture with Gram-positive or Gram-negative bacteria, respectively •Positive clinical or laboratory screen	Peripheral blood	•Identified up-regulated miR-15a and miR-16 in neonatal sepsis, with AUC values of 0.85 and 0.86, respectively, for neonatal sepsis diagnosis	•The miRNAs identified were not validated in large-scale studies in multiple neonatal units
		**Controls** •Blood collected from patients with upper respiratory infection or pneumonia			
Yu et al., 2016	miRNA microarray and qRT-PCR	Neonates (*n* = 5) and adults (*n* = 5) for isolation of majority of leukocyte subpopulations; and neonates (*n* = 31) and adult (*n* = 19) for isolation of pDC **Experimental design** •Human umbilical cord blood was collected from healthy mothers at time of elective Cesarean section or normal spontaneous delivery •Cells were separated and left unstimulated or stimulated with stimulatory agents including LPS	Cord blood	•Identified decreased miRNA let-7b-5p expression in cord blood leukocytes •Let-7b-5p miRNA inhibits LPS-induced IL-6 and TNF-α production in monocytes	•Neonatal leukocyte responses were compared to adults instead of age-matched controls, with a small neonatal sample size used particularly for isolation of leukocyte subpopulations •Only specific leukocyte populations were used for analyses instead of whole blood •The leukocyte responses were assessed using cord blood from neonates born to healthy mothers and may not be reflective of miRNA-mediated regulation of immune responses in neonates with culture-proven bacterial sepsis, the population most in need of improved sepsis management
Kim et al., 2015	Magnetic multiplexed nano-biosensor platform for MMP-7 and EpCAM	20 preterm neonates (24–35 weeks gestation); with sepsis (*n* = 5), NEC (*n* = 10) and controls (*n* = 5). **Sepsis** •Positive blood culture •Absence of uniform serum CRP elevation **NEC** •Pneumatosis intestinale in pathognomonic abdominal radiographic result **Controls** •Absence of sepsis and NEC	Peripheral blood plasma	•MMp7/EpCAM ratio has high diagnostic accuracy for differentiating infants with NEC or sepsis from control infants with AUC values of 1.00 and 0.96 respectively	•Pilot study with small sample size and no power analysis performed •Authors concede that controls may yield some false negatives for sepsis or NEC •The biosensor platform not yet validated in large-scale multicenter studies
Ng et al., 2010	MALDI-TOF MS and protein chip arrays	**Biomarker discovery cohort** 74 very preterm neonates (< 32 weeks gestation), with sepsis/NEC (*n* = 37) or non-sepsis (*n* = 37) **Case-control validation cohort** 80 neonates; with sepsis/NEC (*n* = 40) and non-sepsis (*n* = 40) **Prospective validation cohort** 104 preterm neonates (< 35 weeks gestation); with sepsis/NEC (*n* = 42), probable clinical sepsis (*n* = 13) and non-sepsis (*n* = 49)	Peripheral blood plasma	•ApoSAA score capable of differentiating infants with sepsis and NEC from control infants **Case-control validation cohort** •ApoSAA score showed highest diagnostic performance for infants with sepsis and NEC at a 0.75 cut-off value; with 90% sensitivity and 95% specificity **Prospective validation cohort** •ApoSAA score at 0.75 cut-off value showed 84% specificity and 89% sensitivity	•Mix of bacterial and fungal infections precludes comparisons with studies containing only bacterial sepsis •The algorithm not yet evaluated in large-scale multicenter bacterial sepsis studies
		**Sepsis cases** •Positive blood culture for bacterial or fungal infection **NEC cases** •Stage II or above in Bell's classification **Non-sepsis cases** •Negative blood culture with definitive diagnosis unrelated to sepsis/NEC **Probable clinical sepsis** •Negative blood culture •Presenting >3 clinical signs and symptoms •Evidence of hematologic or metabolic derangement •Strong circumstantial background of sepsis based on clinical course and laboratory results			
Buhimschi et al., 2011	**Discovery phase** Two-dimensional gel electrophoresis and MS **Validation phase** Immunoassay, western blotting and latent-class analysis	180 preterm neonates (< 37 weeks gestation) total for discovery and validation phases **Discovery phase** 6 neonates; with clinical EOS (*n* = 3) and gestational age-matched control (*n* = 3) **Clinical EOS cases** •Positive blood culture •Cord blood IL-6 > 90 pg/mL •Histological chorioamnionitis stages II (chorionic inflammation) or III (full-thickness inflammation of both amnion and chorion) **Controls** •Negative blood culture •Cord blood IL-6 < 9 pg/mL •No histological chorioamnionitis **Validation phase** 174 neonates; with clinical EOS (*n* = 45) and no clinical EOS (*n* = 129). **Clinical EOS** •Positive blood culture **and/or** positive hematological indices (≥2 of following: absolute neutrophil count of < 7,500 or >14,500 cells/mm^3^; absolute band count >1,500 cells/mm^3^; immature/total neutrophil (I:T) ratio >0.16; platelet count < 150,000 cells/mm) **No clinical EOS** •Negative blood culture •Negative hematological indices	Cord blood serum	**Discovery phase** •Identified 19 proteins involved in immunity and defense, protease/extracellular matrix, and transfer/carrier pathways **Validation phase** •Hp & HpRP significantly increased in EOS	•Need to be validated in a large-scale multicenter cohort to ensure Hp & HpRP can be used across different neonatal units to improve EOS diagnosis
Mickiewicz et al., 2013	NMR Spectroscopy	140 patients; of which neonates (*n* = 7), infants (*n* = 47), toddlers (*n* = 54) and school age children (*n* = 32) had either septic shock (*n* = 60), SIRS (*n* = 40) or were healthy controls (*n* = 40) **Septic shock** •PRISM III-APS score with median of 3.5 (3.25–3.75) •Median PCT of 1.7 ng/mL **SIRS** •PRISM III-APS score with median of 24 (12–27). •Median PCT of 3.3 ng/mL **Healthy controls** •Exclusion criteria used: any acute illness, recent use of anti-inflammatory medicine (within 2 weeks), a recent febrile illness (within 2 weeks) or any history of acute or chronic disease associated with inflammation	Peripheral blood serum	•Identified increased levels of lactate, glucose, creatinine, 2-oxoisocaproate, 2-hydroxyisovalerate and 2-hydroxybutyrate; and decreased threonine, acetate, 2-aminobutyrate and adipate in sepsis •Identified increased levels of glucose, 2-hydroxybutyrate and glycerol; and decreased threonine, taurine, suberate, serine, pyruvate, ornithine, methionine, lactate, isoleucine, hypoxanthine, glycine, glutamate, alanine, and adipic acid in septic shock	•Small sample size of neonates, with no age-matched controls •Mixed case definition for septic shock consisting of Gram-positive, Gram-negative and polymicrobial infection •The metabolites identified need to be validated in large-scale multicenter study consisting of “a larger cohort of critical ill patients”
Fanos et al., 2014	GC-MS and NMR	25 neonates (< 35 weeks mean gestation); with sepsis (*n* = 9) and healthy controls (*n* = 16) **Sepsis** •Received diagnosis of sepsis **Healthy controls** •Not diagnosed with sepsis and considered healthy	Urine	•Identified increased concentrations of glucose, lactate and acetate; and decreased ribitol, ribonic acid, pseudouridine, 2,3,4-trihydroxybutanoic acid and 3,4,5-trihydroxypentanoic acid in sepsis	•No criteria listed for case definitions •Small sample size with no power analysis conducted •Identified biomarkers not yet validated in large-scale multicenter studies

*Apo, Apolipoprotein; AUC, area under the curve; CRP, C-reactive protein; EOS, early-onset sepsis; EpCam, epithelial cell adhesion molecule; GC-MS, gas-chromatography mass-spectrometry; Hp, haptoglobin; HpRP, haptoglobin-related protein; IL, interleukin; LOS, late-onset sepsis; LPS, lipopolysaccharide; MALDI-TOF, matrix assisted laser desorption ionization time of flight; miRNA, micro-ribonucleic acid; MMP, matrix metalloproteinase; MS, mass spectrometry; NEC, necrotizing enterocolitis; NMR, nuclear magnetic resonance; pDC, plasmocytoid dendritic cell; PRISM III-APS, pediatric risk of mortality III-acute physiology; qRT-PCR, quantitative reverse-transcriptase polymerase chain reaction; SAA, serum amyloid A; SIRS, systemic inflammatory response syndrome; TNF, tumor necrosis factor; VLBW, very low birth weight*.

Smith et al. analyzed blood samples from preterm and term infants taken when investigated for suspected infection using microarray. The study identified a 52-gene network comprising of genes from innate, adaptive and metabolic pathways that could distinguish bacterial infections from uninfected infants with 98% accuracy. This combined immune-metabolic network performed better compared to individual gene sets (65–84%) (Smith et al., 2014). Cernada et al showed that microarray-based gene expression profiling could discriminate between very low birth-weight infants with bacterial sepsis and controls, with good overall sensitivity (100%) but lower specificity (68%). The differences observed between septic and non-septic controls were associated with 554 differentially expressed genes mainly linked to tumor necrosis factor and cytokine signaling (Cernada et al., 2014). A microarray-based study by Wynn et al. demonstrated that infants with EOS or LOS had different transcriptomes to non-septic infants. However, early and late septic responses differed significantly and were associated with postnatal age at the time of sepsis. These findings underscore the importance of controlling for postnatal age in neonatal sepsis transcriptome studies (Wynn et al., 2015).

Recent transcriptomic studies have also explored the diagnostic and prognostic potential of micro-ribonucleic acids (miRNA) in neonatal sepsis (Table 1). miRNAs are more stable than messenger (m)RNA and increasing evidence supports their importance in sepsis pathophysiology and potential as sepsis markers (Wang et al., 2015; Inal et al., 2016).

Chen et al. identified 10 miRNAs significantly altered during neonatal sepsis in preterm and term infants using microarray, which were confirmed using quantitative real-time reverse transcription-polymerase chain reaction (qRT-PCR). The identified miRNAs were linked to genes and proteins involved in pathogen recognition, inflammation, immune cell activation, release of pro-inflammatory cytokines and apoptosis (Chen et al., 2014). Wang et al. assessed the utility of adult sepsis miRNA biomarkers (miR-15a/15b/16/223) using blood samples collected during septic screens from term neonates. qRT-PCR analysis showed only miR-15a and miR-16 was significantly up-regulated in neonatal sepsis patients compared to controls, with higher area under the curve (AUC) values compared to miR-15b and miR-223. Both miR-15a and miR-16 were found to play a pivotal role in regulating lipopolysaccharide (LPS)-induced inflammatory responses during sepsis (Wang et al., 2015). Separately, in LPS stimulated neonatal leukocytes derived from cord blood of infants delivered by healthy mothers, miRNA let-7b-5p expression was found to be significantly lower compared to adults (Yu et al., 2016).

The mRNA and miRNA studies reviewed observed transcriptional profile differences between infected and control cases that were consistently linked to functions of the innate immune system. Collectively, these studies demonstrate the potential of microarray-based approaches to identify new gene signatures for improved pathophysiological understanding of neonatal sepsis. DNA microarrays, though limited by the number of probes available, remain the most common method to determine transcriptional expression level changes during neonatal sepsis for both mRNA and miRNA (Skibsted et al., 2013; Cernada et al., 2014; Chen et al., 2014; Smith et al., 2014; Wynn et al., 2015).

RNA-Seq is emerging as a powerful tool for transcriptome-wide profiling that is independent of pre-identified probe sequences, thus allowing discovery of novel gene transcripts to generate sepsis-related gene signatures (Chaussabel et al., 2010). Although there have been no published studies using RNA-Seq to identify transcriptional signatures for diagnosis of neonatal sepsis to date, the method has shown potential in adult studies. Pena et al. found that adult sepsis was associated with an endotoxin tolerance signature that was useful in differentiating true from suspected sepsis prior to clinical sepsis confirmation and organ dysfunction prognosis (Pena et al., 2014). RNA-Seq allows for hypothesis-free assessment of the transcriptome for unbiased discovery-based identification of gene signatures associated with neonatal sepsis. Presently, the feasibility of translating NGS-based approaches into bedside tools for neonatal sepsis remains unknown. The challenges faced are both downstream, where extensive sample processing is required for nucleic acid extraction and library preparation for sequencing; and upstream, data storage (up to 150GB per whole-genome sequencing experiment).

RNA-Seq also involves computationally intensive analysis pipelines requiring specialized bioinformatics skills. One possible solution to this is to reduce the dimensionality of the data obtained from patients, focusing only on the minimal identified gene signature required for adequate diagnostic performance (Costa, 2014; Pena et al., 2014; Smith et al., 2014; Tebani et al., 2016b). Such genes could be detected using more rapid approaches such as nanostring and qRT-PCR. Nanostring allows detection of up to 800 different transcripts in a single reaction and is not influenced by pipetting errors, nonspecific enzymatic reactions or reference gene instability, enabling more accurate measurements of gene expression. However, it is limited by the time involved from sample collection to quantitation (16–48 h) compared to qRT-PCR (within 2 h). qRT-PCR has been routinely used for validation of gene expression in microarray and NGS-based sepsis studies, showing promise for clinical translation in childhood leukemia studies and possibility of using a minimal identified gene signature for neonatal sepsis diagnosis in the clinical setting. This approach provides a cost-efficient, rapid, less computationally and bioinformatically intensive alternative to measure gene signature expression for neonatal sepsis diagnosis (Hoffmann et al., 2006; Chaussabel et al., 2010; Chen et al., 2014; Tsang et al., 2017).

### Proteomics of sepsis samples

Overall, no single protein biomarker has emerged with sufficient sensitivity, specificity and reproducibility to accurately diagnose sepsis. This has prompted the need to move from single protein markers, such as CRP or procalcitonin, to the use of panels of biomarkers to improve diagnostic performance (Ludwig and Hummon, 2017). Proteomic studies on body-fluids and tissues in both human adults and animal models assessing the mechanism of both adult and neonatal sepsis have previously been reviewed (Cao and Robinson, 2014; Delanghe and Speeckaert, 2015). This section focuses on recent proteomic studies aimed at identifying biomarkers for human neonatal sepsis.

Necrotizing enterocolitis (NEC) shares similar manifestations with neonatal sepsis, including increased CRP levels, making it difficult to distinguish from sepsis. Several proteins have been identified as potential candidates to differentiate both sepsis and/or NEC from uninfected neonates (Table 1). Kim et al. employed a multiplexed nano-biosensor proteomic platform on plasma samples from 20 preterm neonates to formulate a ratio of protein levels based on matrix metalloproteinase (MMP)-7 and epithelial cell adhesion molecule (EpCAM) concentrations. The MMP-7/EpCAM ratio differentiated NEC from sepsis and healthy controls with high diagnostic accuracy, although the study was limited by a small sample size and lack of information on how neonates were diagnosed for sepsis (Kim et al., 2015). Ng et al. assessed plasma samples from preterm infants with or without NEC/sepsis using ProteinChip array and matrix assisted laser desorption ionization-time of flight (MALDI-TOF) mass spectrometry (MS). The protein concentrations identified from diagnostic proteomic peaks were measured by immunoassay. Multivariate logistic regression analysis determined Apolipoprotein (Apo)C2 and serum amyloid A (SAA) as the most promising markers based on immunoassay concentrations, and were used to construct an ApoSAA score that was capable of differentiating sepsis/NEC cases from non-sepsis/NEC cases. Downstream validation in separate case-control and prospective cohort studies of preterm infants showed high diagnostic performance. Further confirmation in large multicenter trials will be necessary before the ApoSAA score can be translated for use to diagnosis sepsis/NEC cases (Ng et al., 2010). Separately, Buhimschi et al. profiled the serum proteome from venous cord blood of preterm neonates with and without EOS. Gel and MS-based proteomics identified 19 proteins with differential abundance between cases and controls. Downstream validation involving neonates with and without EOS showed that Haptoglobin (Hp) and Haptoglobin-related protein (HpRP) immunoreactivity were significantly elevated in both clinical and culture-confirmed EOS neonates, and that a combination of Hp & HpRP, IL-6 and neonatal hematological indices could improve the clinical EOS diagnosis (Buhimschi et al., 2011).

Together, these proteomic studies highlight the potential to use a panel of biomarkers for more effective diagnosis. Importantly, the use of MS-based technologies for proteomic profiling in these studies underscore the capacity to discover new protein biomarker combinations through a hypothesis-free unbiased approach (Ng et al., 2010; Buhimschi et al., 2011). Compared to traditional hypothesis-driven approaches, where proteins are pre-selected for analysis, MS-based omics can screen thousands of protein abundances and post-translational modifications in a single acquisition, allowing unprecedentedly wide coverage for discovery of novel biomarkers for panel construction. Proteomic biomarkers identified through this approach have potential to be translated into use on bench-top mass spectrometers with optimized and validated assays that are ready for clinical use, allowing timely analysis of multiple proteins in very low abundances close to the bedside (Honour, 2003; Rifai et al., 2006; Ludwig and Hummon, 2017). However, translating a MS-based approach for diagnosis of neonatal sepsis in the clinic has not been assessed. Instrumentation costs will need to be reduced and a standardized protocol for sample collection, preparation, processing, analysis and reporting that is not time-intensive (< 12 h) needs to be developed before MS-based technologies can be feasibly utilized by the bedside (Tebani et al., 2016a). Multiplex immunoassays can be developed from identified multi-biomarker panels and performed using available technologies such as Luminex™. A point-of-care protein-microarray device for quantification of multiple serum proteins using minimal sample volume from neonates has previously been developed, supporting potential to translate validated multi-protein biomarker panels for bedside diagnosis (Buchegger et al., 2012; Tighe et al., 2015).

### Metabolic phenotyping of septic infants

Sepsis induces hypoxia, oxidative stress, and an increased demand for energy resulting in both glucose metabolism and oxidative metabolism of fatty acids; necessitating the monitoring of metabolome dysregulations during neonatal sepsis. Metabolomics can characterize thousands of intermediate to low molecular-weight carbohydrates, amino acids, lipids and other molecules generated by the interaction between host genome, the gut microbiome and environment. It is thus a useful tool to investigate metabolic perturbations related to neonatal sepsis for identification of novel biomarkers. The mainstream analytical technologies for metabolic profiling are nuclear magnetic resonance (NMR) and MS connected to capillary electrophoresis, gas chromatography (GC) or liquid chromatography (LC) separation methods. Whilst NMR enjoys relatively fast and straightforward metabolite annotation and preservation of samples, MS is more sensitive and can detect metabolites with lower abundance. Clinical and pharmaceutical applications of metabolomics in newborns and infants has been reviewed previously (Sumner et al., 2007; Antonucci et al., 2012; Dessì et al., 2014; Fanos et al., 2014). This section focuses on metabolomic studies in neonatal sepsis (Table 1).

Mickiewicz et al. used H-NMR analysis of serum samples from a mixed pediatric cohort consisting of neonates and children up to 11 years of age. Several metabolites, including lactate, glucose, adipate, 2-hydroxybutyrate and threonine were differentially regulated between septic and non-septic patients across age groups, and also distinguished those with systemic inflammatory response syndrome (SIRS). The outcome models designed using orthogonal partial least square discriminant analysis (OPLS-DA) was predictive of mortality in pediatric patients with septic shock (Mickiewicz et al., 2013). Fanos et al. profiled the metabolome of non-invasively collected urine samples from preterm septic newborns and healthy controls using H-NMR and GC-MS, and found increased concentrations of several metabolites including glucose and lactate but decreased concentrations of metabolites including ribitol, pseudouridine and 2-ketogluconic acid. Urine samples from sepsis patients also had differential levels of acetone ketone bodies, likely due to the hypermetabolic responses occurring during sepsis. Interestingly, OPLS-DA of the samples showed clear separation of control and sepsis samples, with ability to discriminate EOS from LOS (Fanos et al., 2014).

Overall, despite different sample types used, both metabolomic studies consistently found increased levels of glucose and lactate in septic patients. The inclusion of both metabolites with other biomarkers such as ribitol, pseudouridine, adipate, 2-hydroxybutyrate and threonine suggests potential to develop a panel of stable early predictors for neonatal sepsis. These studies demonstrate the ability to conduct hypothesis-free screening of the metabolome using metabolomics-based technologies (NMR and MS) to discover novel compositions of metabolites for neonatal sepsis diagnosis (Mickiewicz et al., 2013; Fanos et al., 2014). In particular, coupling of MS analyzers with different separation methods (GC or LC) for acquisitions could improve sensitivity, specificity, chemical coverage and dynamic range for untargeted metabolite discovery from various biological samples (May and McLean, 2016; Tebani et al., 2016b). With clear evidence of metabolic disturbances caused by sepsis or infection, metabolites possess high potential to serve as biomarkers or predictors for sepsis and infection during the neonatal period. Currently, only a limited number of small-scale studies have been carried out, and translation of identified metabolite biomarker panels for bedside diagnosis of neonatal sepsis remains unknown (Mickiewicz et al., 2013; Fanos et al., 2014). MS-based analyses remain time intensive and costly, requiring extensive sample processing in addition to specialized hardware and software for spectral data acquisition, preprocessing and data analysis. Despite this, MS-based technologies have routinely been used for screening of inborn errors of metabolism in newborns with commercial companies working to develop metabolite-based biomarker diagnostic tests, paving the way for future translation of a quick and accurate metabolite-based biomarker test for neonatal sepsis diagnosis (Nagana Gowda and Raftery, 2013; Tebani et al., 2016b).

### Toward precision medicine for neonatal sepsis

Despite limited research in the field of neonatal sepsis to date and limitations within each study (Table 1), available data highlights potential of omics-based approaches to interrogate sepsis pathophysiology for the discovery of novel biomarkers and diagnostic signatures for sepsis. Investigating a septic event at multiple levels, such as across the transcriptional and metabolic response, and at different times during sepsis should capture novel features that account for interactions between genes and biomolecules in a systems network. This can also identify the dynamic changes between networks of genes and biomolecules from different systems, facilitating the discovery of novel dynamical network biomarkers for neonatal sepsis. Identification of dynamical network biomarkers, unlike traditional markers such as the use of a single protein concentration, may allow precise stratification of patients by disease phenotype (such as severity), thereby allowing better prognosis and targeted use of therapeutics (Christaki and Giamarellos-Bourboulis, 2014; Li and Chen, 2014).

**Figure 1:**
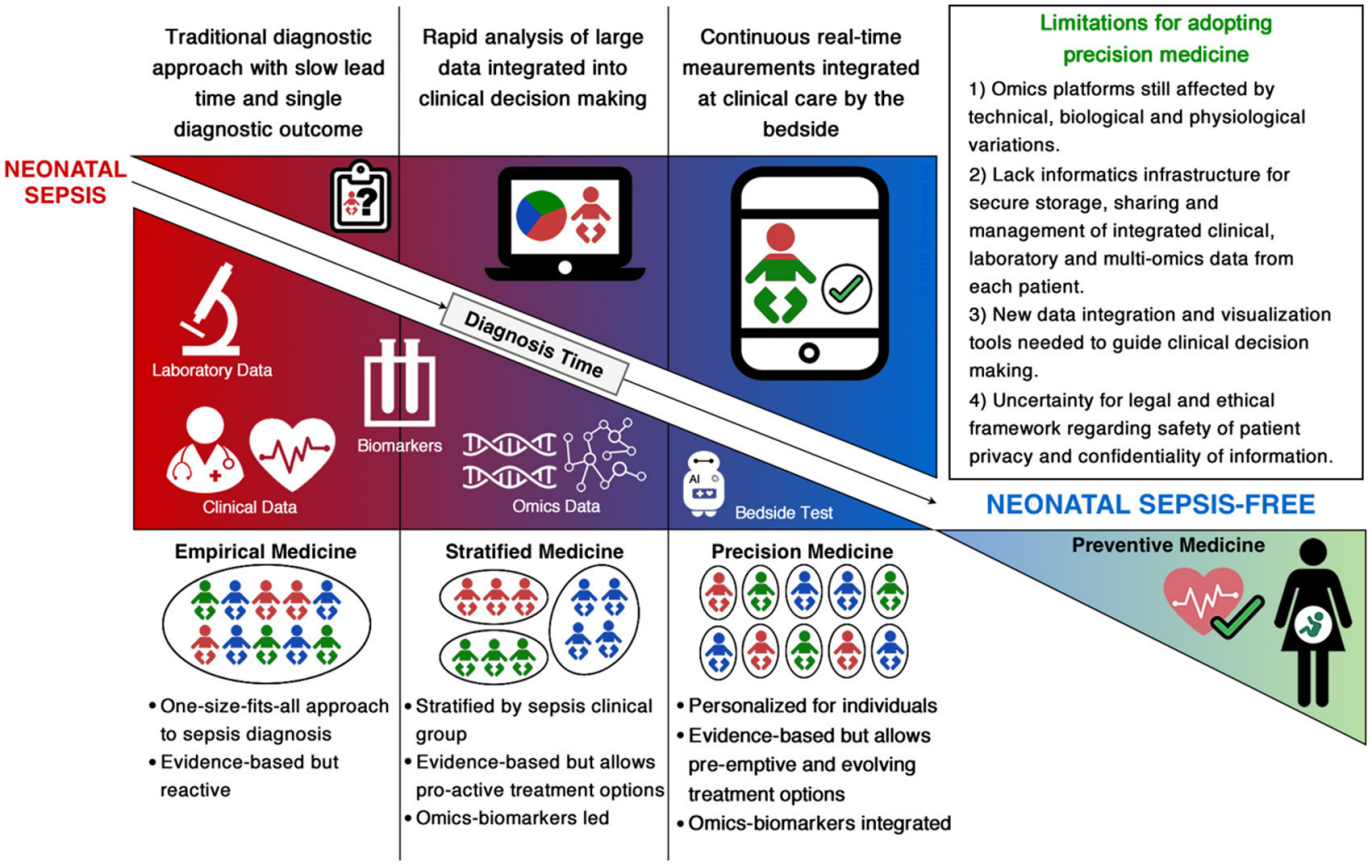
Summary of current and future approaches for diagnosis of neonatal sepsis.

The shift from empirical evidence-based medicine to stratified, omics-led medicine for neonatal sepsis remains in its infancy and faces several challenges (Figure 1). Firstly, neonatal sepsis is a heterogeneous clinical syndrome without a consensus definition, therefore available studies have used differing case definitions (Table 1). Multiple factors contribute to heterogeneity of disease, including the specific pathogen involved (type, load and site of invasion) and the maturational state and capacity of the host immune system (influenced by gestational and postnatal age and associated comorbidities). The inability to stratify patients by disease state based on a clear and consistent definition hinders our ability to compare findings between studies, impeding progress of validating identified biomarkers or gene signatures that can universally improve diagnostic and prognostic tests for neonatal sepsis (Chen et al., 2014; Wynn, 2016). Secondly, we lack representative animal models to investigate and validate neonatal sepsis biomarkers and investigate the dynamics of sepsis pathophysiology. Thirdly, novel biomarkers discovered will need to be selected from an appropriate biological sample that can be pragmatically translated for clinical use (Thongboonkerd, 2013). Given the limited number of studies that have identified biomarkers capable of translating into a bench-side test for neonatal sepsis, analyzing a wide range of longitudinal samples, including whole blood, plasma, serum and urine would be the most comprehensive way of finding novel biomarkers and diagnostic gene signatures (Willis and Lord, 2015). The robustness of the identified markers will need to be validated in appropriately powered multicenter studies to account for instrumental, technical, biological and physiological variations. Finally, whilst our ability to generate omics-based data for neonatal sepsis has significantly improved in the last 10 years with the reducing cost and increasing speed of high-throughput technologies, it has led to a bottleneck with data analysis and interpretation.

Theoretically, discovery of biomarkers and/or sepsis signatures through integration of multi-omic data has potential to stratify neonates with sepsis for improved treatment and prognosis. However, despite increasing access to multi-omics profiles, our ability to analyze and interpret high-dimensional data remains limited by our understanding of the complex interactions between the genome, transcriptome, proteome and metabolome. The complexity of sepsis pathophysiology, heterogenic nature of clinical data, along with bioinformatical and statistical limitations hinder our ability to integrate multi-omic data for interpretation of relationships between “networks” and “networks of networks” (Vucic et al., 2012; Li and Chen, 2014; Alyass et al., 2015). Data storage and management solutions that can integrate each patient's clinical records, laboratory results and omics data need to be developed and implemented before we can transition into precision medicine by the bedside. These solutions will need to be robust enough to deal with the gigabytes of multiple omic platform-specific data, from a range of biological sample types, and across varying instrumental technologies (Gullapalli et al., 2012; Tebani et al., 2016b).

As we move from stratified medicine into the era of precision medicine, the gap between biological science and medicine needs to be bridged in order to translate a patient-specific bedside test for neonatal sepsis. This will require open collaborations and knowledge sharing across multiple disciplines from laboratory scientists to statisticians, computational biologists and clinicians. Further, the translation will require investment into informatics infrastructures that can meet the computationally intensive pipelines for integrative multi-omics, clinical and laboratory data analytics and visualization (Figure 1). Additionally, the legal and ethical framework ensuring confidentiality and privacy of patient information will need to be established. The storage and secure sharing of data through cloud computing solutions will also need to be addressed in order to provide informative digital health reports that guide clinicians for personalized management of sepsis in neonates (Costa, 2014; Alyass et al., 2015; Willis and Lord, 2015; Tebani et al., 2016b).

The use of precision medicine is highly relevant for neonatology. The heterogeneous nature of neonatal sepsis means that our current approaches to diagnosis are too simplistic and imprecise to identify affected individuals accurately (Wynn, 2016). The nature of neonatal medicine, with its highly intensive and frequent monitoring of infants, often over protracted periods and from birth, makes it ideal for translation to the precision medicine setting. Neonatal precision medicine will allow a more predictive and preventive approach, where septic infants can be identified ahead of clinical disease phenotype expression, ensuring prompt and effective antibiotic use. Ultimately, the era of precision medicine for neonatal sepsis, built on the platforms of omics technologies, holds promising potential to ensure quicker and more accurate diagnosis of neonatal sepsis for personalized treatment and improve prognosis of septic neonates (Thongboonkerd, 2013; Christaki and Giamarellos-Bourboulis, 2014).

## Author contributions

SN, PJ, and TM researched the topic and wrote draft manuscripts. TS, PS, and AC contributed to discussions and drafting of the manuscript.

### Conflict of interest statement

The authors declare that the research was conducted in the absence of any commercial or financial relationships that could be construed as a potential conflict of interest.

## References

[B1] Alyass A. Turcotte M. Meyre D. (2015). From big data analysis to personalized medicine for all: challenges and opportunities. BMC Med. Genomics 8:33. 10.1186/s12920-015-0108-y 26112054 PMC4482045

[B2] Antonucci R. Pilloni M. D. Atzori L. Fanos V. (2012). Pharmaceutical research and metabolomics in the newborn. J. Matern. Fetal. Neonatal. Med. 25(Suppl. 5), 22–26. 10.3109/14767058.2012.714634 23025765

[B3] Arboleya S. Sanchez B. Milani C. Duranti S. Solis G. Fernandez N.. (2015). Intestinal microbiota development in preterm neonates and effect of perinatal antibiotics. J. Pediatr. 166, 538–544. 10.1016/j.jpeds.2014.09.041 25444008

[B4] Bateman S. L. Seed P. C. (2010). Procession to pediatric bacteremia and sepsis: covert operations and failures in diplomacy. Pediatrics 126, 137–150. 10.1542/peds.2009-3169 20566606 PMC3142627

[B5] Buchegger P. Sauer U. Toth-Szekely H. Preininger C. (2012). Miniaturized protein microarray with internal calibration as point-of-care device for diagnosis of neonatal sepsis. Sensors 12, 1494–1508. 10.3390/s120201494 22438722 PMC3304124

[B6] Buhimschi C. S. Bhandari V. Dulay A. T. Nayeri U. A. Abdel-Razeq S. S. Pettker C. M.. (2011). Proteomics mapping of cord blood identifies haptoglobin “switch-on” pattern as biomarker of early-onset neonatal sepsis in preterm newborns. PLoS ONE 6:e26111. 10.1371/journal.pone.0026111 22028810 PMC3189953

[B7] Camacho-Gonzalez A. Spearman P. W. Stoll B. J. (2013). Neonatal infectious diseases: evaluation of neonatal sepsis. Pediatr. Clin. North Am. 60, 367–389. 10.1016/j.pcl.2012.12.003 23481106 PMC4405627

[B8] Cao Z. Robinson R. A. S. (2014). The role of proteomics in understanding biological mechanisms of sepsis. Proteomics Clin. Appl. 8, 35–52. 10.1002/prca.201300101 24339042

[B9] Cernada M. Serna E. Bauerl C. Collado M. C. Perez-Martinez G. Vento M. (2014). Genome-wide expression profiles in very low birth weight infants with neonatal sepsis. Pediatrics 133, e1203–1211. 10.1542/peds.2013-2552 24709930

[B10] Chaussabel D. Pascual V. Banchereau J. (2010). Assessing the human immune system through blood transcriptomics. BMC Biol. 8:84. 10.1186/1741-7007-8-84 20619006 PMC2895587

[B11] Chen J. Jiang S. Cao Y. Yang Y. (2014). Altered miRNAs expression profiles and modulation of immune response genes and proteins during neonatal sepsis. J. Clin. Immunol. 34, 340–348. 10.1007/s10875-014-0004-9 24668300

[B12] Chirico G. Loda C. (2011). Laboratory aid to the diagnosis and therapy of infection in the neonate. Pediatr. Rep. 3:e1. 10.4081/pr.2011.e1 21647274 PMC3103129

[B13] Christaki E. Giamarellos-Bourboulis E. J. (2014). The beginning of personalized medicine in sepsis: small steps to a bright future. Clin. Genet. 86, 56–61. 10.1111/cge.12368 24579691

[B14] Costa F. F. (2014). Big data in biomedicine. Drug Discov. Today 19, 433–440. 10.1016/j.drudis.2013.10.012 24183925

[B15] Delanghe J. R. Speeckaert M. M. (2015). Translational research and biomarkers in neonatal sepsis. Clin. Chim. Acta. 451, 46–64. 10.1016/j.cca.2015.01.031 25661089

[B16] Dessì A. Liori B. Caboni P. Corsello G. Giuffre M. Noto A.. (2014). Monitoring neonatal fungal infection with metabolomics. J. Matern. Fetal. Neonatal. Med. 27(Suppl, 2), 34–38. 10.3109/14767058.2014.954787 25284175

[B17] Dong Y. Speer C. P. (2015). Late-onset neonatal sepsis: recent developments. Arch. Dis. Child. Fetal Neonatal Ed. 100, F257–263. 10.1136/archdischild-2014-306213 25425653 PMC4413803

[B18] Fanos V. Caboni P. Corsello G. Stronati M. Gazzolo D. Noto A.. (2014). Urinary (1)H-NMR and GC-MS metabolomics predicts early and late onset neonatal sepsis. Early Hum. Dev. 90(Suppl. 1), S78–S83. 10.1016/S0378-3782(14)70024-6 24709468

[B19] Fanos V. Van den Anker J. Noto A. Mussap M. Atzori L. (2013). Metabolomics in neonatology: fact or fiction? Semin. Fetal Neonatal Med. 18, 3–12. 10.1016/j.siny.2012.10.014 23195852

[B20] Flores M. Glusman G. Brogaard K. Price N. D. Hood L. (2013). P4 medicine: how systems medicine will transform the healthcare sector and society. Per. Med. 10, 565–576. 10.2217/pme.13.57 25342952 PMC4204402

[B21] Gullapalli R. R. Desai K. V. Santana-Santos L. Kant J. A. Becich M. J. (2012). Next generation sequencing in clinical medicine: challenges and lessons for pathology and biomedical informatics. J. Pathol. Inform. 3:40. 10.4103/2153-3539.103013 23248761 PMC3519097

[B22] Hoffmann K. Firth M. J. Beesley A. H. de Klerk N. H. Kees U. R. (2006). Translating microarray data for diagnostic testing in childhood leukaemia. BMC Cancer 6:229. 10.1186/1471-2407-6-229 17002788 PMC1609180

[B23] Honour J. W. (2003). Benchtop mass spectrometry in clinical biochemistry. Ann. Clin. Biochem. 40, 628–638. 10.1258/000456303770367216 14629800

[B24] Inal C. Tanriover M. D. Dayangac Erden D. (2016). Novel transcriptional biomarkers for diagnosis and prognosis of sepsis. Acta Med. Cordoba 5, 11–18.

[B25] Kim D. Fu C. Ling X. B. Hu Z. Tao G. Zhao Y.. (2015). Pilot application of magnetic nanoparticle-based biosensor for necrotizing enterocolitis. J. Proteomics Bioinform. Suppl. 5, 1–6. 10.4172/jpb.S5-002 26798207 PMC4718576

[B26] Kuppala V. S. Meinzen-Derr J. Morrow A. L. Schibler K. R. (2011). Prolonged initial empirical antibiotic treatment is associated with adverse outcomes in premature infants. J. Pediatr. 159, 720–725. 10.1016/j.jpeds.2011.05.033 21784435 PMC3193552

[B27] Li Y. Chen L. (2014). Big biological data: challenges and opportunities. Genom. Proteom. Bioinformatics 12, 187–189. 10.1016/j.gpb.2014.10.001 25462151 PMC4411415

[B28] Ludwig K. R. Hummon A. B. (2017). Mass spectrometry for the discovery of biomarkers of sepsis. Mol. Biosyst. 13, 648–664. 10.1039/C6MB00656F 28207922 PMC5373965

[B29] May J. C. McLean J. A. (2016). Advanced multidimensional separations in mass spectrometry: navigating the big data deluge. Annu. Rev. Anal. Chem. 9, 387–409. 10.1146/annurev-anchem-071015-041734 27306312 PMC5763907

[B30] Mickiewicz B. Vogel H. J. Wong H. R. Winston B. W. (2013). Metabolomics as a novel approach for early diagnosis of pediatric septic shock and its mortality. Am. J. Respir. Crit. Care Med. 187, 967–976. 10.1164/rccm.201209-1726OC 23471468 PMC3707368

[B31] Nagana Gowda G. A. Raftery D. (2013). Biomarker discovery and translation in metabolomics. Curr. Metab. 1, 227–240. 10.2174/2213235X113019990005 27134822 PMC4848463

[B32] Ng P. C. Ang I. L. Chiu R. W. Li K. Lam H. S. Wong R. P.. (2010). Host-response biomarkers for diagnosis of late-onset septicemia and necrotizing enterocolitis in preterm infants. J. Clin. Invest. 120, 2989–3000. 10.1172/JCI40196 20592468 PMC2912182

[B33] Pena O. M. Hancock D. G. Lyle N. H. Linder A. Russell J. A. Xia J.. (2014). An endotoxin tolerance signature predicts sepsis and organ dysfunction at initial clinical presentation. EBioMedicine 1, 64–71. 10.1016/j.ebiom.2014.10.003 25685830 PMC4326653

[B34] Polin R. A. (2012). Management of neonates with suspected or proven early-onset bacterial sepsis. Pediatrics 129, 1006–1015. 10.1542/peds.2012-0541 22547779

[B35] Rifai N. Gillette M. A. Carr S. A. (2006). Protein biomarker discovery and validation: the long and uncertain path to clinical utility. Nat. Biotechnol. 24, 971–983. 10.1038/nbt1235 16900146

[B36] Schmutz N. Henry E. Jopling J. Christensen R. D. (2008). Expected ranges for blood neutrophil concentrations of neonates: the Manroe and Mouzinho charts revisited. J. Perinatol. 28, 275–281. 10.1038/sj.jp.7211916 18200025

[B37] Sharma D. Farahbakhsh N. Shastri S. Sharma P. (2017). Biomarkers for diagnosis of neonatal sepsis: a literature review. J. Matern. Fetal Neonatal Med. 31, 1646–1659. 10.1080/14767058.2017.1322060 28427289

[B38] Skibsted S. Bhasin M. K. Aird W. C. Shapiro N. I. (2013). Bench-to-bedside review: future novel diagnostics for sepsis - a systems biology approach. Crit. Care 17:231 10.1186/cc12693 24093155 PMC4057467

[B39] Smith C. L. Dickinson P. Forster T. Craigon M. Ross A. Khondoker M. R.. (2014). Identification of a human neonatal immune-metabolic network associated with bacterial infection. Nat. Commun. 5:4649. 10.1038/ncomms5649 25120092 PMC4143936

[B40] Sumner L. W. Amberg A. Barrett D. Beale M. H. Beger R. Daykin C. A.. (2007). Proposed minimum reporting standards for chemical analysis Chemical Analysis Working Group (CAWG) Metabolomics Standards Initiative (MSI). Metabolomics 3, 211–221. 10.1007/s11306-007-0082-2 24039616 PMC3772505

[B41] Tebani A. Abily-Donval L. Afonso C. Marret S. Bekri S. (2016a). Clinical metabolomics: the new metabolic window for inborn errors of metabolism investigations in the post-genomic era. Int. J. Mol. Sci. 17. 10.3390/ijms17071167 27447622 PMC4964538

[B42] Tebani A. Afonso C. Marret S. Bekri S. (2016b). Omics-based strategies in precision medicine: toward a paradigm shift in inborn errors of metabolism investigations. Int. J. Mol. Sci. 17:E1555. 10.3390/ijms17091555 27649151 PMC5037827

[B43] Thongboonkerd V. (2013). The promise and challenge of systems biology in translational medicine. Clin. Sci. 124, 389–390. 10.1042/CS20120565 23083321

[B44] Tighe P. J. Ryder R. R. Todd I. Fairclough L. C. (2015). ELISA in the multiplex era: potentials and pitfalls. Proteomics Clin. Appl. 9, 406–422. 10.1002/prca.201400130 25644123 PMC6680274

[B45] Tsang H. F. Xue V. W. Koh S. P. Chiu Y. M. Ng L. P. Wong S. C. (2017). NanoString, a novel digital color-coded barcode technology: current and future applications in molecular diagnostics. Expert Rev. Mol. Diagn. 17, 95–103. 10.1080/14737159.2017.1268533 27917695

[B46] van Karnebeek C. D. M. Wortmann S. B. Tarailo-Graovac M. Langeveld M. Ferreira C. R. van de Kamp J. M.. (2018). The role of the clinician in the multi-omics era: are you ready? J. Inherit. Metab. Dis. 41, 571–582. 10.1007/s10545-017-0128-1 29362952 PMC5959952

[B47] Vucic E. A. Thu K. L. Robison K. Rybaczyk L. A. Chari R. Alvarez C. E.. (2012). Translating cancer 'omics' to improved outcomes. Genome Res. 22, 188–195. 10.1101/gr.124354.111 22301133 PMC3266027

[B48] Wang X. Wang X. Liu X. Wang X. Xu J. Hou S.. (2015). miR-15a/16 are upreuglated in the serum of neonatal sepsis patients and inhibit the LPS-induced inflammatory pathway. Int. J. Clin. Exp. Med. 8, 5683–5690. 26131152 PMC4483976

[B49] Willis J. C. Lord G. M. (2015). Immune biomarkers: the promises and pitfalls of personalized medicine. Nat. Rev. Immunol. 15, 323–329. 10.1038/nri3820 25814400

[B50] Wynn J. L. (2016). Defining neonatal sepsis. Curr. Opin. Pediatr. 28, 135–140. 10.1097/MOP.0000000000000315 26766602 PMC4786443

[B51] Wynn J. L. Guthrie S. O. Wong H. R. Lahni P. Ungaro R. Lopez M. C.. (2015). Postnatal age is a critical determinant of the neonatal host response to sepsis. Mol. Med. 21, 496–504. 10.2119/molmed.2015.00064 26052715 PMC4607623

[B52] Xia J. Gill E. E. Hancock R. E. (2015). NetworkAnalyst for statistical, visual and network-based meta-analysis of gene expression data. Nat. Protoc. 10, 823–844. 10.1038/nprot.2015.052 25950236

[B53] Yu H. R. Hsu T. Y. Huang H. C. Kuo H. C. Li S. C. Yang K. D.. (2016). Comparison of the functional microRNA expression in immune cell subsets of neonates and adults. Front. Immunol. 7:615. 10.3389/fimmu.2016.00615 28066425 PMC5165026

